# Expanding the Paradigms of Plant Pathogen Life History and Evolution of Parasitic Fitness beyond Agricultural Boundaries

**DOI:** 10.1371/journal.ppat.1000693

**Published:** 2009-12-24

**Authors:** Cindy E. Morris, Marc Bardin, Linda L. Kinkel, Benoit Moury, Philippe C. Nicot, David C. Sands

**Affiliations:** 1 INRA, Unité de Pathologie Végétale UR407, Montfavet, France; 2 Department of Plant Sciences and Plant Pathology, Montana State University, Bozeman, Montana, United States of America; 3 Department of Plant Pathology, University of Minnesota, St. Paul, Minnesota, United States of America; The Fox Chase Cancer Center, United States of America

## Introduction

How do pathogens, whether they parasitize plants or animals, acquire virulence to new hosts and resistance to the arms we deploy to control disease? The significance of these questions for microbiology and for society at large can be illustrated by the recent worldwide efforts to track and limit the emergence of human transmissible strains of swine and avian influenza virus and of multidrug-resistant lines of human pathogenic bacteria, and to restrain the spread of Ug99, a strain of stem rust of wheat. Recent research in medical epidemiology has elucidated the impact of pathogen ecology in environmental reservoirs on the evolution of novel or enhanced pathogen virulence. In contrast, the evolution of virulence in plant pathogens has been investigated from a predominantly agro-centric perspective, and has focused overwhelmingly on evolutionary forces related to interactions with the primary plant host. Here, we argue that current concepts from the field of medical epidemiology regarding mechanisms that lead to acquisition of novel virulence, biocide resistance, and enhanced pathogenic fitness can serve as an important foundation for novel hypotheses about the evolution of plant pathogens. We present numerous examples of virulence traits in plant pathogenic microorganisms that also have a function in their survival and growth in nonagricultural and nonplant habitats. Based on this evidence, we make an appeal to expand concepts of the life history of plant pathogens and the drivers of pathogen evolution beyond the current agro-centric perspective.

## Paradigms of Evolution of Virulence in Human “Environmental Pathogens”

The classification of diseases in terms of their epidemiology is a useful starting point for a comparison of plant and human pathogens [1]. In medical epidemiology, anthroponoses are diseases transmitted among humans that have no other known reservoirs for multiplication. Typhoid fever, smallpox, and certain venereal diseases are examples. Zoonoses, such as rabies, lyme disease, severe acute respiratory syndrome (SARS), and avian and swine influenzas, are transmitted to humans from living animals. Sapronoses are diseases transmitted to humans from environmental reservoirs where the pathogen thrives saprophytically. These habitats include soil, water, and decaying plant and animal matter. Examples include Legionnaire's disease, cholera, aspergillosis, and the emerging epidemics of melioidosis (*Burkholderia pseudomallei*). Human pathogens with saprophytic phases or residing in environmental reservoirs are also referred to as “environmental pathogens” [2]–[6].

Studies of virulence factors of human pathogens in environmental reservoirs have begun to reveal the importance of alternate hosts, of dual-use virulence factors, and in general of how environmental habitats can select for traits that confer enhanced fitness as human pathogens. For example, interactions with microbial eukaryotes seem to have led to the acquisition of traits useful for pathogenicity to mammalian cells. Numerous environmental pathogens, including *Cryptococcus neoformans*, *Legionella* spp., *Chlamydophila pneumoniae*, *Mycobacterium avium*, *Listeria monocytogenes*, *Pseudomonas aeruginosa*, and *Francisella tularensis*, might have acquired virulence traits via their resistance to predation by amoebae. This resistance, associated with the ability to grow inside the amoebae—which are essentially alternate hosts—has likely led to the selection of traits conferring survival in macrophages [7]. Resistance to macrophages involves the capacity of the bacteria to resist or debilitate the macrophage's phagosomes and to multiply in the cytoplasm. Many of the traits essential for virulence to humans likewise seem to play roles in adaption to the environments where the organisms are saprophytes (Table 1). These traits have dual roles in environmental and parasitic fitness and are thus referred to as “dual-use traits”. Melanins, siderophores, and the capacity to form biofilms are among the frequently cited examples. *C. neoformans* provides one of the richest examples of dual-use traits. This fungus, frequently found in soils that contain high levels of bird guano and in association with certain plants, causes meningoencephalitis. A nonexhaustive list of its dual-use traits includes capsule formation and production of melanin, laccase, phospholipase, proteases, and ureases [8]. In the environment these traits contribute to survival and in human hosts they contribute to the capacity of *C. neoformans* to avoid host resistance mechanisms and to attack host tissue. Microbial efflux pumps have also evolved dual uses. These transport systems are used for managing toxic compounds in the environment of the microorganism and can have a broad spectra of activity leading to multidrug resistance among environmental microorganisms [9]. Human activities resulting in the disposal of a wide range of chemical products into the environment, including household cleaners that contain the broad spectrum antimicrobial triclosan, may be inadvertently exacerbating the abundance of multidrug-resistant bacteria [10].

**Table 1:** Examples of putative dual-use traits related to pathogenic and environmental fitness of human pathogens.

Organism	Trait or Gene	Role in Pathogenic Fitness	Role in Environmental Fitness	Reference
*Vibrio cholera*	Toxin co-regulated pilus	Virulence factor in humans	Biofilm formation on chitin	[59],[60]
*Legionella pneumophila*	Eukaryotic-like proteins that mimic cellular functions of eukaryotic proteins; type II and type IV secretion systems, surface proteins involved in attachment, secreted effectors	Virulence factors in macrophages	Parasitism and multiplication in protozoa	[61]
*Burkholderia cenocepacia*	Quorum-sensing regulatory system	Regulation of virulence factors implicated in “cepacia syndrome”	Regulation of factors involved in nematode killing	[62]
*Yersinia pestis*	Extracellular polysaccharide production linked to the action of heme storage gene (*hms*) products	Transmission to the human host and protection from the action of leukocytes	Colonization of flea esophagus via biofilm formation	[63]
*Cryptococcus neoformans, Alternaria fumigatus*	Melanins	Protects microbial cells against phagocytosis	Protection against oxidation	[24]
*Alternaria flavus, Histoplasma capsulatum, Aspergillus fumigatus, A. nidulans* and numerous bacteria	Siderophores	Virulence factor in humans	Sequestering iron in the environment	[21]–[23]
*Pseudomonas aeruginosa* and *Stenotrophomonas maltophilia*	Efflux pumps	Intrinsic multidrug resistance	Exclusion of lipophilic toxic compounds from cells	[10],[64],[65]
*Acinetobacter baummannii*	Efflux pumps, genetic promiscuity, exopolysaccharides and biofilm formation, siderophore-like compounds	Multidrug resistance, attachement, stimulation of host inflammation, virulence factor in humans	Exclusion of toxic compounds from cells, resistance to desiccation, sequestering of iron	[66]

Virulence of environmental pathogens has been described as a set of cards, or a diverse set of attributes acquired as a function of the life history of a pathogen and its adaptation to different environments [3],[8]. It is becoming increasingly clear that evolutionary forces outside the context of human–pathogen interactions are responsible for the acquisition and maintenance of some virulence factors [11]. Genomics and phylogenetics are revealing the evolutionary link between, for example, commensal strains of *Escherichia coli* and modern pathogens such as enterohaemorrhagic strains of this species (such as O157). The mechanisms proposed to explain how these commensals have become pathogens are grounded in their ecology and life histories, culminating in the notion of ecological evolution (“eco-evo”) [11]. The eco-evo approach to understanding the emergence of pathogens gives credence, from the perspective of genomics, to evolutionary and adaptive scenarios that are surmised from a thorough understanding of the ecology and life history of pathogens.

## Links between Plant Pathogenicity, Adaptation to Biotic and Chemical Stress, and Key Vital Functions

At present, epidemiological classifications of plant diseases are based on the interaction of the pathogen and the host (biotrophic or necrotrophic, obligate or facultative), on the number of cycles of propagule production (mono- and polycyclic diseases), on the importance of latency in symptom expression, and on the role of vectors, but there is no formalized equivalent of “sapronoses”. Nevertheless, numerous plant pathogens are present in diverse nonagricultural habitats or survive saprophytically in agricultural contexts. These include a range of bacteria, fungi, and stable viruses (a nonexhaustive list of examples is presented in Table 2). A striking characteristic of many of the virulence factors of these plant pathogens is that they are linked to—or are in themselves—traits critical to adaptation to the nonplant environment, as will be illustrated below. This provides a compelling reason to adopt a holistic view of the life history and evolution of plant pathogens, to move beyond the traditional borders of agriculture and the presumed “primary” plant host. Adaptation to biotic and abiotic stresses, within or outside of agricultural habitats, likely plays as important a role in the evolution of parasitic fitness of plant pathogens as it does for human pathogens.

**Table 2:** Examples of plant pathogens reported to thrive in nonagricultural habitats or to survive saprophytically in agricultural contexts in the absence of host plants.

Species	Nonagricultural Habitats or Substrates Where Microbe Has Been Detected	Putative Factors Conducive to Survival	References
**Bacteria**			
*Burkholderia cepacea*	Ubiquitous in soils and waters and associated habitats	Unusually large genome harboring genes for a multitude of traits related to ecological fitness including the capacity to use a large spectrum of carbon sources	[67]
*Dickyea* spp. including *D. chrysanthemi* and *Pectobacterium carotovorum* (formerly *Erwinia chrysanthemi and E. carotovora*)	Oceanic aerosols, soils, alpine rivers, and other surface water, snow	Capacity of pectolytic bacteria to obtain nutrients from rotting plant material and to use a wide range of carbon sources; cell surface properties than foster condensation of water vapor; growth and survival as a facultative anaerobe	[68]–[71]
*Pantoea agglomerans*	Fecal matter, soil, surface waters	This bacterium is generally an opportunistic plant pathogen that is normally a fit saprophyte	[72],[73]
*Pseudomonas syringae*	Clouds, snow rain, epilithic biofilms, wild alpine plants (substrates linked to the water cycle)	Biofilm formation; production of toxins and siderophores; survival of freezing	[74],[75]
*Rhodococcus fascians*	Soil, ice, polar seawater, lesions on animals, rinds of cheese	Sexual promiscuity favoring acquisition of diverse plasmid-borne traits; capacity to shift metabolic pathways as a function of food base	[76]–[78]
*Streptomyces* spp.	Ubiquitous in soil and water	Production of a diverse array of degradative enzymes critical to saprophytic lifestyle; capacity to produce a wide range of antibiotics important in species interactions; resistant to many antibiotics	[29]
**Fungi**			
*Alternaria* spp.	Most *Alternaria* species are common saprophytes; found in soil or decaying plant tissues and atmospheric aerosols	Derive energy as a result of cellulytic activity. Production of toxic secondary metabolites. Production of melanin protecting against environmental stress or unfavorable conditions (extreme temperatures, UV radiation and compounds secreted by microbial antagonists).	[79],[80]
*Aspergillus* spp.	Marine and terrestrial habitats, soil; associated with insects, humans, and other animals	Production of toxins including aflatoxins; production of siderophores and degradative enzymes (pectinases, proteases)	[81]–[84]
*Cladosporium* spp.	Soil; atmospheric aerosols	Carbohydrate-binding protein modules (LysM effectors). No other suppositions found in the literature.	[18],[79],[81],[83]
*Fusarium* spp.	Soil; extreme saline soil habitats; marine and fluvial habitats	Production of defense-related metabolites (antibiotics, trichotecenes, mycotoxins…) and of siderophores; vigor in competitive use of foods, ability to colonize a wide range of substrates	[81], [83], [85]–[89]
*Leptosphaeria maculans*	Can survive as a saprobe for many years on debris	Maintains numerous genes required for saprophytic life (for nutrient acquisition, competition with soil microflora), necrotrophic parasitism via toxins and degradative enzymes	[90]
*Mucorales: Mucor* spp., *Rhizopus* spp.	Soil and a variety of organic substrates; marine habitats including insect cadavers	Production of siderophores (by *Rhizopus*)	[81]–[83],[91]
*Pythium* spp. (nonobligate parasitic oomycetes)	Soil and water	No suppositions found in the literature	[92]
*Penicillium* spp.	Soil, sediment-rich subglacial ice; atmospheric aerosols	Production of toxins and siderophores	[79], [81]–[83],[93]
**Viruses**			
Tomato mosaic virus	Clouds, glacial ice, soil of pristine forests	Overall stability of tobamoviruses	[94]–[96]

As illustrated above, traits that confer fitness in response to biotic and abiotic environmental stress can have dual-use as virulence factors in human pathogens. Toxins and toxin transport systems (including efflux pumps, in particular) are among the common adaptations for antagonizing and defending against the co-inhabitants of a habitat. In plant pathogens, the transport systems for toxins and antimicrobials can have broad spectrum activity, leading to resistance to agricultural fungicides and also contributing to virulence [12]. Genes coding for wide spectrum efflux pumps are present in the chromosomes of all living organisms [9]. The efflux pump BcAtrB of *Botrytis cinerea* confers resistance to antimicrobials produced by soil and plant microflora (2,4-diacetylphloroglucinol and phenazine antibiotics) [13],[14] and also to the fungicide fenpiclonil and the plant defensive phytoalexin resveratrol [15]. The transporter ABC1 from *Magnaporthe grisea* protects the fungus against azole fungicides and the rice phytoalexin sakuranetin [12]. Numerous plant pathogenic bacteria, including *Erwinia amylovora*, *Dickeya* spp. (formerly the multiple biovars of *E. chrysanthemi*), and *Agrobacterium tumefaciens*, also produce efflux pumps that are involved in their resistance to plant antimicrobials (reviewed by Martinez et al. [9]). Toxins themselves can have a broad spectrum of action. For example, mycotoxins, well known for their human and animal toxicity, have broad spectrum activity and are thought to have evolved as a defense against predators (nematodes) and antagonists (other microorganisms) [16]. One family of these, the trichothecenes, contributes significantly to the virulence of many *Gibberella* (*Fusarium*) species [17].

Adaptation to biotic stress also implicates systems for the detection or inhibition of arms of aggression used by co-inhabitants. Recent work on fungi suggests that systems to detect enzymes that degrade fungal cell walls are also deployed as virulence factors. Lysin motifs (LysMs) are carbohydrate-binding protein modules that have been found in mammalian and plant pathogenic fungi as well as in saprophytes [18]. Bolton et al. [19] demonstrated that the LysM protein Ecp6 acts as a virulence factor in the plant pathogenic fungus *Cladosporium fulvum*. As virulence factors they may suppress host defenses by sequestrating chitin oligosaccharides that are known to act as elicitors of plant defense responses [19] and also as activators of host immune responses in mammals [20]. de Jonge and Thomma [18] suggest that these proteins may also have a role in the protection of saprophytic fungi against chitinase-secreting competitor microbes or mycoparasites.

Protection against abiotic stress can involve molecules that have also become virulence factors. Siderophores [21]–[23] and various pigments including melanins [24] are virulence factors in some human pathogens. Siderophores contribute to resistance to oxidative stress and sequestering iron when it is rare in the environment. In the plant pathogens *Alternaria brassicicola*, *Cochliobolus* spp., *Fusarium graminearum* [25], and *M. grisea* [26], siderophores or their precursors are virulence factors. Melanins offer protection from extreme temperatures, UV radiation, and antimicrobials. In the plant pathogens *M. grisea* and *Colletotrichum* spp., melanins are also virulence factors via their essential role in the formation of tissue-penetration structures such as appressoria [17]. In many cases, toxins and siderophores are produced by nonribosomal peptide synthase or polyketide synthase pathways. These pathways, widely distributed in the microbial world, are highly adaptable and have given rise to a wide range of compounds with a plethora of activities, including many of pharmaceutical importance [27]. HC-toxin of *Cochliobilus carbonum*, victorin in *C. victoriae*, and T-toxin in *C. heterostrophus* are products of these pathways [28]. The key virulence factor of *Streptomyces* spp., thaxtomin [29], and the multitude of host-specific and nonspecific toxins in *Pseudomonas syringae* pathovars [30] are also produced by these pathways.

The capacity to detect changes in conditions of the abiotic environment has also become part of the virulence factors of some plant pathogens. For example, to detect changes in environmental conditions, organisms exploit two-component histidine kinase complexes. These are key elements of the machinery for signal sensing, allowing bacteria, yeasts, fungi, and plants to adapt to changing environments. In the plant pathogen *B. cinerea*, one of its multiple histidine kinases, BOS1, not only mediates osmosensitivity and resistance to fungicides, but is also essential for formation of macroconidia and expression of virulence [31].

Recognition and understanding of the full complexity of the life history of plant pathogens will enhance our capacity to evaluate the diversity and intensity of environmental stresses that microorganisms face and will contribute novel hypotheses concerning the role of environmental stresses in the evolution of pathogenicity. Stress is considered to play an important role in adaptive evolution in general, in particular via its effect on mutation rates [32]. For certain fungi and bacteria, including plant pathogens, stress increases the activity of transposable elements [33]–[35] and induces the SOS response and other systems involved in the modification or repair of DNA [32]. Mutations can target the ensemble of the microbial genome. However, it has been suggested that adaptation of bacteria to multiple stresses can lead, in particular, to the acquisition of virulence factors and to the emergence of pathogenic variants [36].

Adaptation to specific habitats—which involves adapting to a particular ensemble of biotic and abiotic parameters—could also influence the evolution of parasitic fitness. Available examples focus on soil-borne and rhizosphere microorganisms. The rhizosphere is a dynamic soup whose chemistry changes as plants grow, die, and degrade. Chemicals in the rhizosphere are food substrates and means of communication, antagonism, and collaboration among microorganisms, among plants, and between plants and microorganisms. To decompose dead plant material and recycle carbon, microorganisms have developed a range of cell wall–degrading enzymes, without which our planet would be quite encumbered by the accumulation of tissue from dead plants. Pectolytic, cellulolytic, and lignolytic enzymes are also well-known pathogenicity factors [37]–[39]. To hone the efficiency of these enzymes *in planta*, pectinolytic fungi are adept at modulating the surrounding pH. *Alternaria*, *Penicillium*, *Fusarium* spp., and *Sclerotinia sclerotiorum* also exploit these pH changes to enhance the action of these enzymes as virulence factors [40]. *Streptomyces* spp. are considered quintessential soil inhabitants. Their ability to degrade biopolymers, including cellulose and chitin, contributes greatly to nutrient cycling, and their vast array of antimicrobials contributes to survival and microbial communication in soil [29]. Some *Streptomyces* species are pathogenic to root crops and to potatoes in particular. A recently discovered virulence factor in *Streptomyces*, a saponinase homologue [29], may be the result of adaptation to the rhizosphere. Saponins are plant glycosides that contribute to resistance against fungi and insect herbivores. Bacteria, and especially Gram-positive bacteria, can also be sensitive. Saponins are also exuded from the roots of some plant species where they have allelopathic as well as antimicrobial activity [41],[42].

Key vital functions, housekeeping functions, and basic life cycle processes should also be considered for their potential to give rise to pathogenicity factors. Traits fundamental to fitness and survival in general can confer or enhance pathogenic fitness. In plant pathogenic bacteria these include flagella, motility, lipo- and exo- polysaccharides, O-antigens, fimbriae, mechanisms for iron acquisition and for quorum sensing, toxin production, cell wall–degrading enzymes, and resistance to oxidative stress [43]. Motility, for example, is essential to dispersal and for attaining new resources. In *Ralstonia solanacearum* it is also essential for early stages of plant invasion and colonization during pathogenesis [44]. In the fungus *Aschochyta rabiei*, kinesins that are essential for polarized growth and transport of organelles are suspected to be a virulence factor [45]. An F-box protein of *Giberrella zeae* has been reported to be involved in sexual reproduction and in pathogenicity [46]. The enzymes that allow fungi to detoxify compounds resulting from plant defense mechanisms are probably also simply means of acquiring nutrients [47]. For example, detoxification of tomatine in tomatoes by *Septoria lycopercici* and by *Fusarium oxysporum* f. sp. *lycopersici* is achieved by the deployment of glycosyl hydrolases by these fungi; *Gaeumannomyces graminis* detoxifies avenacins in oats via a beta-glucosidase [28]. Another example of adaptation of basic cellular functions into pathogenicity factors concerns elicitins. Elicitins are part of one of the most highly conserved protein families in the *Phytophthora* genus and are widespread throughout *Phytophthora* species. Elicitins of *P. infestans* induce hypersensitivity in plants. Recent work from Jiang and colleagues [48] suggests that a primary function of elicitins is the acquisition of sterols from the environment.

## Toward New Paradigms about the Evolution of Plant Pathogenicity: The Roles of Dual-Use Traits and Exaptation

How can we make sense of the processes that have led to the wide variety of pathogenicity factors in plant pathogens and that continue to drive the evolution of pathogens? Bacterial plant pathogens are particularly illustrative of the differences in suites of secretion systems [43],[49],[50],[51] and of effectors [50],[51],[52],[53],[54],[55] among members of different genera, species, or strains of the same species that attack plants. Effectors are proteins secreted by plant pathogens that modulate plant defense reactions, thereby enabling the pathogen to colonize the plant tissues. It is tempting to wonder if the effectors and secretion systems have critical roles in fitness elsewhere other than in association with the host plant. The examples listed above that describe traits that play roles in both environmental fitness and virulence to plants provide a compelling incentive to expand our paradigms concerning the forces that drive evolution of plant pathogenicity. The evolutionary forces that have been described to date for plant pathogens [56] need to be extended beyond the current agro-centric paradigm.

To expand this paradigm we propose that the life cycles and life histories of plant pathogens be reconsidered. Studies of pathogen ecology, evolution, and life history should include the full range of habitats and reservoirs these organisms can inhabit. This in turn will permit testing a range of novel hypotheses about the role of ecological contexts—other than direct interaction with host plants—as forces of evolution. In Table 3 we propose some such hypotheses. For example, rates of mutation and of transposition of insertion sequences or of transposable elements including phages might be different when a microorganism inhabits nonagricultural habitats (biofilms, lake water, or inert surfaces exposed to UV, for example) than when it colonizes plants. The consequences of these mutations for pathogenicity might in turn be markedly different than for fitness in nonagricultural habitats. Likewise, the formation of spores or aggregates that can be released into the air and their survival over long distances might be highly influenced by the nature of the reservoir that the pathogen colonizes, resulting in direct effects of habitat on gene flow. Furthermore, the biotic and abiotic stresses endured in nonagricultural habitats might exert positive selection for adaptive survival traits that have dual-use as virulence factors as illustrated in the examples above. These questions are clearly pertinent for pathogens that are not obligate biotrophs. However, the complexity of the biotic and abiotic environment perceived by obligate biotrophs during colonization of plants (powdery mildews on leaf surfaces inhabited by other microorganisms, for example) or during their dissemination (survival in air or in association with vectors) are also likely to exert selection independent of that due to the host plant genotype per se. These are only some of the ways in which environmental parameters other than the host plant are expected to have a marked influence on the diversification of plant pathogens.

**Table 3:** Novel hypotheses to be tested concerning the impact of substrates other than host plants on the evolutionary potential of plant pathogens.

Evolutionary Force^a^	Novel Hypothesis Arising from Expanded Paradigms about the Evolution of Plant Pathogenicity Concerning:
Mutation	***Modifications of the genome***.
	Relative to its association with cultivated plant hosts, association of the pathogen with a given nonagricultural substrate leads to:
	• a significantly greater overall mutation rate.
	• a greater rate of transposition of insertion sequences or of transposable elements.
	• more frequent mutations or transpositions that target genes involved in pathogenicity.
	• a higher probability of acquisition of alien nucleic acids.
	• genetic exchange with more phylogenetically diverse microbes.
Genetic drift	***Effective population size***.
	The effective sub-population size of a pathogen associated with a given nonagricultural (or nonplant) substrate is significantly different from that for sub-populations from cultivated host plants. This could lead to genetic and/or phenotypic differentiation of sub-populations based on substrate of origin.
Gene flow	***Dissemination***.
	The habitats occupied by the plant pathogen influence the mode(s) of dissemination, thereby influencing the distance of dissemination and the spatial and temporal scales of gene flow.
Mode of reproduction (recombination)	***Genetic recombination***.
	The frequency of recombination (via sexual cycle or other means) varies among strains of plant pathogens as a function of the habitat or substrate.
Selection	***Selective pressures and impact on fitness***.
	Strains of pathogens adapted to a broad range of habitats have the greatest parasitic fitness.

a The evolutionary forces listed here are those that have been considered for plant pathogens in agricultural contexts [56]. These hypotheses concern pathogens with a marked saprophytic phase or for which nonagricultural or nonplant substrates can be a notable reservoir for survival. Reservoirs can include irrigation water, natural waterways and bodies of water, biological vectors (animals, fungi, etc.), abiotic vectors (aerosols, clouds, precipitation), wild plants and weeds, soil, and physical structures in agricultural systems (greenhouse materials, tubing, plastics).

If nonagricultural environments can foster the evolution of traits that contribute to pathogen virulence, other scenarios are also probable where i) crop plants foster the emergence of traits antagonistic to survival outside of agricultural contexts ii) or nonagricultural environments foster the emergence of traits that are detrimental to pathogen virulence in crops. Understanding the prevalence and significance of alternative habitats to pathogen life history is crucial to determining the broad costs of virulence for pathogen fitness. The cost of virulence in terms of fitness in association with plants has been explored extensively for several obligate parasites such as rusts and powdery mildews. Work by Thrall and Burdon [57] has shown clear fitness tradeoffs between pathogen aggressiveness (capacity to induce intense disease symptoms) and dissemination (via intense spore production). For nonobligate pathogens we do not know the cost of fitness outside of agricultural habitats. The interplay between evolutionary forces and habitat has not been explored for plant pathogens and might be a key feature in the emergence of certain diseases.

By expanding our paradigms concerning pathogen life history and the selective forces that drive plant pathogen evolution, we will enhance our understanding of how pathogens survive in the absence of hosts, how and where new pathotypes are likely to emerge, and the significance of natural habitats to agricultural epidemics. Insights will come from fundamental research to identify the mechanisms that drive the evolution of pathogenic traits and to explore the ecological significance of pathogenic traits to microbial fitness apart from the plant host. Distinguishing the role of adaptation *sensu stricto* in the emergence of plant pathogenicity relative to that of exaptation [58], the useful cooptation of phenotypes that have arisen under natural selection due to forces unrelated to interaction with the primary host plant, will yield critical insight into how plant pathogens evolve independently of agricultural practices. A more complete understanding of the forces that drive plant pathogen evolution will be critical to enhancing and diversifying sustainable disease control strategies, and will improve prediction of the conditions that support the emergence of novel pathogens.
